# Glutathione-s-transferase is a minor allergen in birch pollen because of restricted release from hydrated pollen grains

**DOI:** 10.1186/2045-7022-4-S2-O1

**Published:** 2014-03-17

**Authors:** Stephan Deifl, Christian Zwicker, Eva Vejvar, Claudia Kitzmüller, Gabriele Gadermaier, Birgit Nagl, Susanne Vrtala, Gerhard Zlabinger, Peter Briza, Fatima Ferreira, Barbara Bohle

**Affiliations:** 1 Medical University of Vienna, Department of Pathophysiology and Allergy Research, Vienna, Austria; 2 University of Salzburg, Department of Molecular Biology, Salzburg, Austria; 3 Medical University of Vienna, Institute of Immunology, Vienna, Austria

## Background

Recently, proteomic profiling of birch pollen detected a protein homologous to glutathione-S-transferases (GST) in prominent amounts. In mites, cockroach and fungi, GST are relevant allergens. This tempted us to investigate the allergenicity of GST from birch (bGST).

## Methods

bGST was expressed in Escherichia coli, purified and characterized by mass spectrometry. BALB/c mice were immunized with bGST or Bet v 1. Antibody and T cell responses were assessed. 217 sera from birch pollen-allergic patients were tested for IgE-reactivity to bGST by ELISA. The allergenicity of bGST was evaluated with IgE-loaded rat basophil leukaemia cells (RBL) expressing the α-chain of the human receptor FcεRI. Cross-reactivity of IgE between bGST and GST from house dust mite, Der p 8, was assessed with murine and human sera in ELISA. The release kinetics of bGST and Bet v 1 from birch pollen upon hydration were studied by immunoblotting.

## Results

Immunization with bGST induced specific IgE and a Th2-dominated cellular immune response comparably to immunization with Bet v 1. Only 13.4% of birch pollen-allergic patients were sensitized to bGST. In RBL assays bGST induced mediator release. GST from birch and house dust mites did not cross-react. In contrast to Bet v 1, bGST showed a limited and delayed release from hydrated birch pollen grains.

## Conclusion

bGST induces specific IgE in mice but is of limited sensitizing capacity for humans. In contrast to Bet v 1, the release of bGST from hydrated pollen is restricted. Thus, the minor allergenicity of bGST may be explained by a limited exposure of patients to this protein.

